# Integrating Text-Mining and Balanced Scorecard Techniques to Investigate the Association between CEO Message of Homepage Words and Financial Status: Emphasis on Hospitals

**DOI:** 10.3390/healthcare9040408

**Published:** 2021-04-01

**Authors:** Hyung Jong Na, Kun Chang Lee, Seong Tae Kim

**Affiliations:** 1 School of Global Business Administration, Semyung University, Jecheon 27136, Korea; freshna77@semyung.ac.kr; 2 SKK Business School, Sungkyunkwan University, Seoul 03063, Korea; 3 School of Management, Kyung Hee University, Seoul 02447, Korea; goodthink365@naver.com

**Keywords:** homepage words, financial ratio, text mining, balanced scorecard

## Abstract

(1) Background: The Chief Executive Officer’s (CEO’s) message on a hospital’s homepage on the Internet contains various components, such as the hospital’s future vision, promises to customers, availability of upgraded services and public activities. This statement usually includes non-financial information as well as financial information about the corporate entity owning/operating the hospital. In addition, it provides useful information about not only the company’s goals and vision, but also firm performance targets and strategies for the future. This study aims to investigate associations between the CEO’s message and the financial status of the institution. We used the balanced scorecard framework to analyze what content on the hospital’s homepage is related to the hospital’s various financial ratios. (2) Methods: We adopted a text-mining method to extract significantly repeated keywords from the CEO’s message on the hospital’s website. Then, we classified these keywords using a balanced scorecard approach. To examine the relationship between keywords in the CEO’s message and the hospital’s financial ratios, a *t*-test was conducted for the difference in the term frequency divided by inverse document frequency (TF-IDF) mean of the home page contents and its relationship with the views of the balanced scorecard framework. (3) Results: According to our empirical results on 65 samples collected from local hospitals, there are some significant relationships between the qualitative content of the hospital’s homepage and the quantitative financial ratios that indicate profitability, activity, leverage, liquidity, and accumulating reserves for proper business purposes. (4) Conclusions: The introduction section of a homepage is the part most accessible to customers, containing the aims and ideals of the hospital and reflecting the institution’s values and visions. In addition, in the coverage of financial status, the organization can either emphasize financial strength or focus on other areas to divert attention from any weakness shown in the financial information. This study reminds us of the importance of the hospital website’s disclosure, and what can be inferred from the financial status of the hospital. It also highlights the need for reconciliation and harmony between the quantitative data, financial statements, and qualitative data in the CEO’s message. (5) Implications: To the best of our knowledge, this paper is the first research attempting to investigate the relationship between text on the hospital’s homepage and the hospital’s financial ratios using text-mining techniques and the balanced scorecard framework. Hospitals play a crucial role in a country’s welfare and healthcare industry. Nevertheless, in many countries, hospital organizations tend to remain a source of critical fiscal deficits due to ineffective and sloppy management. We expect that the result of this paper can provide hospital managers with useful information to address that situation.

## 1. Introduction

Historically, the Korean government has regarded medical services as a public good. In this sense, the government has aimed to provide medical services as fundamental welfare, so that all the citizens have a right to receive medical care regardless of their financial status. Koreans basically are given medical insurance benefits for most diseases.

Compared to other non-profit organizations, hospitals have higher labor costs and need to invest into expensive medical equipment and related facilities. According to the Korea Medical Law, hospitals have many limitations on running commercial businesses (such as a cafeteria), or in charging rent for use of their facilities. Therefore, Korean hospitals have troubles maximizing their financial competitiveness. Due to the non-profit nature of Korean hospitals, their operating funds mostly consist of medical income and donations.

These two sources of funds are affected by the hospital’s external reputation, image, performance, and value. Hospitals, therefore, try to advertise their strengths, visions, and strategies using many channels. Additionally, they tend to try to hide their weaknesses. For example, if a hospital reports high financial performance, it could result in a negative perception from society, indicating that it pursues profits rather than public value. Therefore, the hospitals have motivation to promote their public interests to society. The size of the hospital (based on total assets) implies indirectly the quality of medical service it is providing. Accordingly, small hospitals should alleviate these negative perceptions from potential customers by underlining operating efficiency. From this point of view, CEOs have an incentive to provide desired information by actively utilizing qualitative information with high discretion in their homepage message, rather than leaning on the quantitative information, which is difficult to manipulate.

To the best of our knowledge, few studies have been accomplished about the relationship between hospitals’ financial information and their CEO’s messaging using a text-mining method. Hence, this study focuses on examining that relationship. The CEO’s message on the hospital’s homepage contains various components, such as the hospital’s future vision, promises to customers, availability of upgraded services and public activities [1].

The CEO tries to convey various types of important information about the firm in the CEO message. That message includes non-financial information as well as financial information about the corporate entity owning/operating the hospital. In addition, it provides useful information about not only the company’s goals and vision, but also firm performance targets and strategies for the future [2].

We expect that some of the qualitative content expressed in the CEO’s message can be construed such that there is a significant relationship with the financial ratio. To analyze this expectation, we used a text-mining technique to convert unstructured data (such as the homepage text) into multiple keywords and classified them into meaningful groups based on a balanced scorecard framework [3].

The specific analysis process of this study was as follows: First, we used a text-mining technique to calculate the term frequency divided by inverse document frequency (TF-IDF) of words from the “About Us” or “CEO Greeting” section of the hospital homepage. The TF-IDF of words indicates how significantly they are repeated in the text contents of the hospital homepage.

Next, we used a balanced scorecard framework to classify words extracted with text mining. The balanced scorecard consists of four perspectives: (a) finance, (b) customer, (c) internal process, and (d) learning and growth. However, since hospitals are non-profit organizations that serve the public, in this study, we replaced financial perspectives with public views. Additionally, if keywords have nothing to do with any of these four points of view, we classified them as neutral words, adding a fifth point of view.

Third, the financial statements disclosed on each hospital’s website were collected. The various financial ratios, such as hospital size, profitability, activity of fixed assets, leverage, liquidity, and accumulating earnings for proper business purposes, were calculated using the data.

Finally, to investigate the relationship between homepage words and the financial ratios, we conducted a *t*-test on the difference of the mean of TF-IDF of the words related to the five perspectives. In the process, we analyzed the difference of the mean of TF-IDF of the words between the high-level and the low-level quintiles within each perspective.

The results of this study are as follows: First, the findings for the public point of view show that the current ratio had a significant difference between more and less frequently used words. Specifically, there was a positive relationship between using words related to the public view more frequently and increasing the current ratio. Secondly, the findings for the customer point of view show that the ratio of accumulating reserves for proper business purposes had a significant difference between more and less frequently used words. In this case, there was a positive relationship between using words related to the customer more frequently and improving the ratio of accumulating reserves for proper business purposes. Thirdly, the findings for the internal process point of view were that the size ratio has a significant difference between more and less frequently used words. There was a negative relationship between using words related to internal process more frequently and increasing the size ratio. In other words, using words related to internal process less frequently has a positive relationship with improving the size ratio. Fourthly, the findings for the learning and growth point of view show that debt ratio, asset turnover ratio, fixed-asset turnover ratio, and the ratio of accumulating reserves for proper business purposes have significant differences between more and less frequently used words. We found a negative relationship between using words related to learning and growth more frequently and an increase in each ratio. Namely, using words related to learning and growth less frequently has a positive association with improving the debt ratio, fixed-asset turnover ratio, asset turnover ratio, and the ratio of accumulating reserves for proper business purposes.

The contributions of this paper are as follows: First, this study used unstructured text data of hospital homepages and conducted empirical tests. By utilizing unstructured data, we expand the range of the hospital and finance research field. Second, by conducting a *t*-test of the relationship between more and less frequently used words on the hospital homepages, we found useful information. Some words have significant relationships to financial ratios. The results of this study supply hospital managers and customers with an understanding of which words can be indicators for financial ratios.

This paper is organized as follows: Prior literature sources related to hospital homepages and hospital finance information is addressed to develop hypotheses in Section 2. Methodology adopted in this study is explained in Section 3. New findings of our empirical analysis are suggested in Section 4, along with discussions and implications. Section 5 has some concluding remarks.

## 2. Review of Prior Literature and Hypothesis Development

### 2.1. Vision Statements

Most hospital homepage introductions mention the vision of the hospital. There has been much research done about vision statements. Quigley (1994) documented that the success of an organization depends upon how well a leader can communicate his or her vision and inspire organization members [4]. Lucas (1998) explained that an effective vision statement should promote growth and development [5]. It also serves to inspire and encourages people to act toward achieving the purpose of the organization. Raynor (1998) found that many executives are convinced of the importance of mission or vision statements [6]. Berson et al. (2001) explained that organizational size relates to vision strength and moderates the association between vision strength and passive leadership style [7]. Gulati et al. (2016) stated that a vision provides an organization with a core goal as well as a bright future [8]. Further, vision is associated with performance outcomes [9].

### 2.2. Hospital Homepage Contents

Homepage contents of hospitals contain not only vision statements but also other meaningful information. Prior studies related to hospital homepage contents explain information utility. Kim and Lee (1999) examined contents and operational situations of homepages [10]. To effectively provide customers with hospital information, they insist that hospital homepages should include various types of content. Lee and Ahn (2012) explained that the university hospital’s website includes relatively more content than other hospitals [11]. They report that the university hospital’s website relatively puts more effort into providing more information to visitors and satisfying customers. Jin et al. (2011) investigated surgery using robotic content presented on the hospital’s website [12]. Their results show that no hospital website commented on risks and that surgical robots overestimate profits. Lee (2013) reported that, in order to improve the hospital’s image, factors such as hospital homepage information and hospital management should be managed well as marketing methods [13].

### 2.3. Hospital Financial Performance

There are prior literature sources related to hospitals and financial performance. Nelson et al. (1992) documented hospital quality as related to financial performance of hospitals [14]. Zeller et al. (1997) outlined how measures of financial factors, such as capital structure, fixed-asset age, working capital liquidity, and fixed-asset efficiency, can be beneficial to hospital boards, policy makers, healthcare financial managers, and other relevant groups [15]. Watkins (2000) found that non-accounting information is highly significant in estimating bond grade [16]. Alexander et al. (2006) reported that the improvement of hospital quality increased hospital organizational performance [17]. Kaissi and Begun (2008) mentioned that the establishment of strategic plans and management participation have positive associations with earlier financial performance [18]. Upadhyay et al. (2015) reported that hospital managers can improve profitability by reducing the length of cash conversion [19]. Dobrzykowski et al. (2016) explained that providing patients with professional service improves patient safety and hospital financial performance [20]. Wang et al. (2018) presented that health information technology expense, capital expense, and information technology operating expense have positive associations with return on assets [21].

### 2.4. Hospital Research Using Text Mining

Hospital research has yet to make full use of text mining. However, the following studies introduced research in the hospital field using text mining. Hahn et al. (2001) analyzed major patterns in medical documents and extracted knowledge using a natural language process [22]. Zhou et al. (2010) mentioned that text mining is one of the most valuable methodologies for researching subfields in data mining [23]. Text mining of patient records can provide valuable information and assist decision making [24]. Yang et al. (2009) utilized text mining to analyze clinical data and predict disease conditions from clinical discharge summaries [25]. They explained the possibility of text mining for more precise prediction of disease status. Kocbek et al. (2016) documented a text-mining approach for detecting marks as positive for some diseases [26]. Using text mining in the medical field can help advance medical technology by making it easier to acquire and utilize new knowledge from medical literature [23].

### 2.5. Hospital Literature Related to Balanced Scorecard Technique

Hospital studies related to the balanced scorecard technique are many, as follows: Stewart and Bestor (2000) designed an integrated performance measurement using balanced scorecard [27]. They developed a balanced scorecard for hospitals consisting of one composite financial performance proxy and 12 nonfinancial performance proxies. These performance proxies are used in terms of actual performance as an expected performance percentage, and an overall performance score is calculated through a subjective weighting scheme. Pink et al. (2001) reported on hospitals in Canada using the balanced scorecard framework [28]. Indicators of balanced scorecard performance were developed in four areas—patient satisfaction, system integration and change, clinical utilization and outcomes, and financial performance and condition. Chen et al. (2006) explained how a balanced scorecard was effective for identifying chances for improvement and underlining existing problems [29]. A balanced scorecard reveals the contribution of hospitals to improve performance in the health system. Zhijun et al. (2014) investigated the current application of the balanced scorecard and the effect upon hospital performance in China [30]. They reported that some Chinese hospitals had used a balanced scorecard system in hospital administration. The findings showed that the balanced scorecard application contributed to improved organizational performance. In addition, they found that the balanced scorecard system is affected by technological quality, operational scope, and comprehensiveness of medical resources. Walker and Dunn (2006) insisted that a balanced scorecard system can measure productivity of hospitals and improve hospital management at reduced cost without loss of quality [31].

### 2.6. Hypothesis Development

Hospital homepage content contains valuable and meaningful information. In particular, introduction sections on homepages offer a summary of important information about the hospital. This content usually mentions vision and performance outcomes [9].

CEOs have an incentive to provide desired information by actively utilizing qualitative information with high discretion in their CEO message rather than leaning on quantitative information, which is difficult to manipulate.

For example, hospitals that report high income or have a lot of current assets that are not conducive to improving the quality of medical services will emphasize their public value through the CEO message in order to break the image of being pro-profits. Hospitals with plans to increase the quality of medical services with new investment will use a lot of patient-related words in communications to improve patient satisfaction. Small-size hospitals will promote operational efficiency by using a lot of words related to internal process to remedy any biased image of low quality of medical service derived from a smaller hospital size. Hospitals with unfavorable financial status, such as insufficient debt financing ability, inefficient usage of fixed assets, or lack of capacity for future medical investment, will underline the effort to increase potential and sustainability by using learning- and growth-related words. In summary, we expect that some of the words on hospital homepages might have significant associations with financial performance.

In this study, to examine the association between introductory words on hospital homepages and financial information, we used the text-mining method and balanced scorecard framework. Text mining plays an important role in obtaining useful implications and meaning from hospital information systems [26]. A balanced scorecard can be the key to achieving strategic goals and operating hospitals effectively [32]. Further, performance measurement using the balanced scorecard system provides an effective way for hospitals to achieve strategic goals [27].

**Figure 1:**
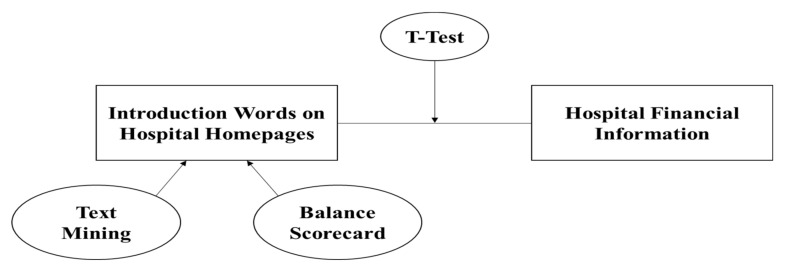
The concept and methodology of this research.

We extracted keywords from introductory content on hospital homepages and classified meaningful words into groups using a balanced scorecard. Then, we conducted a *t*-test to analyze the relationship between those introductory words and financial information. We expected that if we found significant results, introductory text on hospital homages could be indicators of hospital financial information. Below, Figure 1 explains the concept and methodology of this research. Therefore, we developed a hypothesis:

**Hypothesis** **1.** *There might be some significant relationships between the CEO’s message on a hospital’s homepage and financial information.*

Specifically,

**Hypothesis** **1a.** *There might be a significant relationship between the usage of public-related words and high income or financial liquidity.*

**Hypothesis** **1b.** *There might be a significant relationship between the usage of patient-related words and future medical investment.*

**Hypothesis** **1c.** *There might be a significant relationship between the usage of internal process-related words and the hospital’s size.*

**Hypothesis** **1d.** *There might be a significant relationship between the usage of learning- and growth-related words and debt financing ability, activity of fixed assets, or future medical investment.*

## 3. Material and Methods

### 3.1. Sample Selection

#### 3.1.1. Text-Mining Methodology

The sample of this study consists of 65 hospitals that are designated as senior public general hospitals by Korea’s Ministry of Health and Welfare as of 2018. We obtained homepage text data and financial statements. The financial data were collected from the Hospital Accounting Standard System provided by the Korean Health Industry Development Institute. We collected introductory text from “About Us” or “CEO’s Greeting” pages on their websites. To quantify the text, we utilized the text-mining method. Using Java-contained functions, the text was automatically collected. Morpheme division work is done with the codes contained in Java using Porter’s (1980) algorithm. To remove disused words, we conducted POS tagging, a process that classifies words as nouns, verbs, adjectives, adverbs, and so on. Next, we removed verbs, adjectives, adverbs, prepositions, and special marks, which means we used only nouns for the sake of analysis. The symbols that have no lexical meaning, such as sentence marks, were removed. Basic HTML tags also were removed during the text extracting process using a Java-contained function.

Next, we calculated the TF-IDF value for each word used in “About Us” or “CEO’s Greeting” content. The process is based on term frequency information of the Bag of Words model. TF-IDF is a weighted value that allows us to understand how important a given word is in various documents [21]. The reason we did not simply calculate frequency is that frequency itself cannot determine importance in the content. Meaningless words can appear in high frequency and may not play a proper role in distinguishing the difference by their frequent repeat. Therefore, in this study, we used the values of term frequency (TF) and inverse document frequency (IDF).

Words with more than 1% frequency were selected. Including all words as subjects of analysis would make the calculation burdensome, so the words with 1% or more frequency were left in each file. The frequency calculation was conducted by file; therefore, a word left in a specific file might not remain in other files. Our purpose is to find words representing the file’s traits, so high-frequency words could be analyzed. Hence, we aligned the words used in each file and recorded their frequency. As the words appearing in the files are different, we correlated to avoid overlap after they were collected in one place and recorded how many times the words were used in each file. Since the TF-IDF can be different in each file, we recorded the TF-IDF collectively.

#### 3.1.2. Balanced Scorecard Frame

Balanced scorecard as a management accounting methodology has developed and improved over the years as a general management practice [33]. The balanced scorecard is a comprehensive management framework that can readily interact with learning and growth, customers, internal processes, and financial perspectives. The aim of the four perspectives is linked together by cause-and-effect relations [34]. That is, the BSC shows how the interaction is taking place not only from a financial perspective but also from a non-financial perspective (such as customer, process, and learning and growth) by extracting key success factors based on the four perspectives and developing them in the direction of setting the firm’s strategy and goals [35].

We categorized words extracted by text mining, applying the balanced scorecard technique, which is addressed in the Appendix A (refer to Table A1). Many prior studies adopted this method to handle narrative reports [3,36,37,38,39,40]. Balanced scorecard consists of four perspectives: customer, financial, learning and growth, and internal process. However, hospitals can only be established as non-profit organizations. This means hospitals should not aim for financial profit. Therefore, this study changes from the “financial perspective” of the common balanced scorecard to the “public perspective” in accordance with the purpose of the establishment of hospitals.

In summary, we classified the keywords extracted by text mining into four perspectives based on a balanced scorecard framework modified in accordance with the purpose of non-profit hospitals: public (PB), patient (PT), internal process (IP), and learning and growth (LG). However, some words are hard to classify exactly with one of these four perspectives, because they have general or vague connotations. Thus, we classified them as neutral (NT), and we removed those words from the final balanced scorecard perspective in our analysis.

### 3.2. Model Specification

Our research framework is as follows: First, we calculated the mean of TF-IDF for words included in each of the four balanced scorecard perspectives for nonprofit hospitals (TI_PB, TI_PT, TI_IP, and TI_LG). Each mean indicates to what degree a manager focuses on the content of that particular perspective in their homepage text compared with other hospitals. For example, if TI_PB is high, the manager focuses on their public vision in the introductory content. Second, we divided the sample into quintiles by means of the TF-IDF in the balanced scorecard perspective, defining that first and second quintile samples as “high balanced scorecard” and fourth and fifth quintile samples as “low balanced scorecard”. Finally, we compared the mean of financial ratios in the high balanced scorecard group with the low balanced scorecard group by using the *t*-test method.

Our study aimed to examine the relationship between non-financial data and a hospital’s financial information. We utilized financial proxies to assess hospital financial performance, such as profitability, activity, leverage, and liquidity ratio. In addition, Korean hospitals can retain some of their income as an essential business fund (EBF) within a certain limit ranging from 50 to 100%, depending on their establishment form. This money should only be used for medical purposes such as buying medical equipment or building a new ward, and it is classified as long-term liability in a financial statement. Considering this unique characteristic of Korean hospitals, we added several ratios related to the EBF to our analysis.

Commonly used financial information: ratios of profitability, growth, leverage, liquidity, and activity. However, we could not calculate a growth ratio (the change between this year’s revenue and previous year’s revenue to the previous year’s revenue), as we only have one year of data from financial statements. Therefore, in this study, we compared each high balanced scorecard hospital’s financial information with low balanced scorecard hospitals’ information, using profitability, activity, leverage, and retaining an essential business fund.

As described in Table 1, the proxies in our research are size (SIZE), return on assets (ROA), fixed-asset turnover (FTOV), long-term debt ratio (LTLEV), current ratio (CUR), and accumulating reserves for proper business purposes ratio (ACCRV) (under Korean medical law, hospitals are allowed to transfer parts of pre-tax income to temporary reserves to use it for specific purposes, such as acquiring medical equipment or building properties within five years. This transferred income is called “accumulating reserves for proper business purposes”). Each financial ratio is calculated as follows:

## 4. Results

### 4.1. Descriptive Statistics

Table 2 shows the descriptive statistics used in this research. Final hospital samples number 65; according to our balanced scorecard framework, there are four classified variables, and there are six financial ratios used for the *t*-test. TI_PB, TI_PT, TI_IP, and TI_LG are the means of TF-IDF of included words in each of the four balanced scorecard perspectives. The means of TI_PB, TI_PT, TI_IP, and TI_LG in Table 2 are the means of TF-IDF of text on hospital homepages. The TI_PB, TI_PT, TI_IP, and TI_LG means are 0.044, 0.039, 0.042, and 0.044. The standard deviations of TI_PB, TI_PT, TI_IP, and TI_LG are 0.023, 0.021, 0.023, and 0.024.

The mean of ROA is 0.000, standard deviation is 0.103, and median is −0.003. These numbers indicate that most hospitals do not report profit. The mean of ACCRV is 0.535, and the standard deviation is 0.924, which implies that a considerable portion of income is retained as essential business funds.

### 4.2. Research Results

Table 3 shows *t*-test results examining the mean differences of financial ratios. First, in terms of profitability, ROA of a high-PB hospital is higher than that of a low-PB hospital, but not significant (*t*-value = 0.75, *p* = 0.458). The mean of activity and leverage ratios, which are represented by FTOV and LTLEV, are higher in low-PB hospitals, but not statistically significant (*t*-value = −0.21, *p* = 0.834; *t*-value = −1.18, *p* = 0.243). In contrast, the mean difference of CUR is statistically significant at 5%, which indicates that high-PB hospitals have more liquidity than low-PB hospitals. On the other hand, high-PB hospitals’ ACCRV ratio is lower, but not statistically significant, similar to the activity and stability ratios.

In summary, hospitals using many words related to their public vision have higher financial liquidity than other hospitals; however, profitability is not unlike what we expected. Other financial information is almost identical. This result implies that the hospitals with high liquidity emphasize their public value by using more public-related words in the CEO’s message to mask the holding of current assets, which have low contribution to the improvement of medical service quality.

Table 4 presents the *t*-test result examining the mean differences of financial ratios between high-PT hospitals and low-PT hospitals. First, profitability (ROA) of high-PT hospitals is higher than that of low-PT hospitals, but not significant, although there is a relatively high t-value (*t*-value = 1.60, *p* = 0.115). Similar to Table 3, the mean of activity and leverage ratios, which are represented by FTOV and LTLEV, are higher in low-PT hospitals, but not statistically significant (*t*-value = −1.13, *p* = 0.267; *t*-value = −0.21, *p* = 0.838). Unlike Table 3, the liquidity ratio of high-PT hospitals is lower than that of low-PT hospitals, but the mean is not significantly different. However, the gap in the ACCRV ratio between the two is statistically significant at 10% (*t*-value = −1.82, *p* = 0.08). In summary, using many words related to the patient perspective accumulates earnings into proper business purpose funds for future medical investment more than other hospitals. This result indicates that the hospitals with a plan for investing more medical capital should emphasize their effort to improve patient satisfaction by using more patient-related words in the CEO’s message.

Table 5 shows the *t*-test results examining the mean differences of financial ratios between high-IP hospitals and low-IP hospitals. As can be seen in Table 4, no statistical significance of mean difference in any financial ratio exists except for SIZE. This result can be interpreted to mean that hospital managers do not allude to financial information using words related to an internal process perspective in homepage content. On the contrary, the mean of a high-IP hospital’s size is bigger than that of a low-IP hospital’s size, and it is statistically significant at 1% level (*t*-value = −3.06, *p* = 0.004).

In summary, hospitals using many words related to the internal process perspective are smaller in size than other hospitals. This means that small hospitals’ managers try to overcome the relative weakness of the quality of medical service they are providing derived from small scale by emphasizing the efficiency of their internal process rather than communicating other factors on their homepages.

Table 6 shows the *t*-test result examining the mean differences of financial ratio between high-LG hospitals and low-LG hospitals. Unlike IC, there is no significance in the mean difference of hospital size between high- and low-LG hospitals. The means of LTLEV, FTOV, and ACCRV ratios are significantly different between high- and low-LG hospitals at 10, 5, and 10%, respectively. Further, although not statistically significant, the mean difference of the ROA ratio is almost five percentage points between high- and low-LG hospitals.

In summary, hospitals using many words related to the learning and growth perspective have insufficient debt financing capability, inefficient usage of fixed assets, and lack of capacity for future medical investment. As shown in Table 6, financial information of high-LG hospitals is generally more unfavorable than that of low-LG hospitals. Hence, managers of high-LG hospitals are likely to conceal their current low performance, promoting future growth possibilities by writing homepage content related to the learning and growth perspective rather than to existing financial performance.

## 5. Discussion

The empirical results and implications of this paper are as follows: First, low-PB hospitals have low financial liquidity. This means managers of hospitals in financial distress do not focus on public value on their homepages. Second, high-PT hospitals tend to accumulate their earnings to proper business purpose funds. Hospitals focused on patient satisfaction are likely to pursue future medical investment over present performance. Third, there is no significant difference in financial ratios between high-IP and low-IP hospitals, but the size of high-IP hospitals is smaller than that of low-IP hospitals. This result implies that managers of small hospitals account for relative weakness due to size by emphasizing their internal operating efficiency. Finally, high-LG hospitals have relatively low long-term leverage, activity of fixed assets, and accumulating for proper business purposes.

This study has the following limitations: The results of the empirical analysis of this study are difficult to generalize due to the small number of samples. Additionally, since we analyzed only senior general hospitals that met certain criteria, the results could be different when analyzing smaller hospitals.

Despite this limitation, this paper has the following contributions. First, as far as we know, this paper is the first study to investigate the relationship between hospital financial information and the hospital CEO’s message on the homepage using text-mining techniques. In particular, we expect this study to provide a broad understanding of the text-mining methodology in the hospital research area. Second, this study did not rely on any subjective viewpoint, but instead used a balanced scorecard framework to classify the CEO’s message on the hospital’s homepage. Based on these results, we find a significant relationship exists with the hospital’s financial ratios. Namely, this study is expected to contribute to further research development by presenting a new methodology for hospital management research. Additionally, it is expected to remind hospital managers of the importance of qualitative data disclosure online in hospital management.

## 6. Conclusions

We examined the association between the CEO’s message on the hospital’s homepage and the hospital’s financial status. The hospital’s CEO communicates a variety of information to the customer, such as the achievement of the organization’s goals, current status of the organization, and organizational policy. The introductory words of a homepage serve as a summary of important information about the organization [41]. Particularly, the introduction section of a homepage is most accessible to customers, containing the aims and ideals of hospitals and reflecting their values and visions [2]. In addition, in view of financial status, they can either emphasize financial strength or focus on other areas to mask any weakness in the financial information [42].

This study analyzed CEO messages on hospitals’ websites and investigated the message content’s relationship with hospital financial status. To examine this relationship, we used the text-mining method and the balanced scorecard framework. We calculated TF-IDF of each word extracted by the text-mining method, which suggests the importance of words used in the homepage text written by every hospital’s manager. To categorize extracted words into several groups, we adopted a balanced scorecard perspective. Since all hospitals in Korea are nonprofit organizations, we adjusted the financial performance perspective of the balanced scorecard to a public perspective.

The findings of this paper are expected to provide useful information for hospital managers in developing policies. It reminds us of the importance of a hospital’s website disclosures, and what these messages can infer about the financial status of the hospital. It also highlights the need for reconciliation and harmony among quantitative data, financial statements, and qualitative data, as shown in the CEO’s message.

## Figures and Tables

**Table 1:** Variable definition.

Financial Information	Variables	
Hospital size	SIZE	(= natural logarithm of total asset)
Profitability	ROA	(= net income divided by total asset)
Activity	FTOV	(= medical revenue divided by fixed asset)
Leverage	LTLEV	(= long-term debt scaled by total asset)
Liquidity	CUR	(= current asset divided by current liability)
Accumulating reserves for proper business	ACCRV	(= accumulating reserves for proper business divided by pre-tax income)

**Table 2:** Descriptive statistics.

Variable	N	Mean	Std	Min	Q1	Median	Q3	Max
TI_PB	65	0.044	0.023	0.000	0.028	0.043	0.061	0.090
TI_PT	65	0.039	0.021	0.000	0.024	0.036	0.053	0.109
TI_IP	65	0.042	0.023	0.000	0.021	0.043	0.059	0.093
TI_LG	65	0.044	0.024	0.000	0.028	0.044	0.060	0.101
SIZE	65	25.208	1.746	21.416	23.594	25.757	26.357	29.195
ROA	65	0.000	0.130	−0.391	−0.037	−0.003	0.026	0.573
FTOV	65	5.377	8.077	0.000	1.547	2.395	4.614	36.906
LTLEV	65	0.456	0.576	0.000	0.107	0.304	0.589	3.480
CUR	65	1.407	1.182	0.086	0.788	1.049	1.698	7.379
ACCRV	65	0.535	0.924	−2.525	0.000	0.000	1.034	4.306

**Table 3:** The mean difference of TF/IDF on PB between high (1,2) and low (4,5) quintile.

Variable	High			Low			Difference	*t*-Value	Prob-t	Significance
	N	Mean	Std	N	Mean	Std				
SIZE	26	25.186	1.689	26	25.602	1.474	−0.416	−0.95	0.349	
ROA	26	0.017	0.116	26	−0.004	0.077	0.020	0.75	0.458	
FTOV	26	5.250	7.197	26	5.765	10.155	−0.515	−0.21	0.834	
LTLEV	26	0.315	0.337	26	0.439	0.419	−0.125	−1.18	0.243	
CUR	26	1.666	1.226	26	1.084	0.466	0.582	2.22	0.034	**
ACCRV	26	0.479	0.950	26	0.540	0.594	−0.061	−0.28	0.780	.

Note: ** denotes 0.05 significance level.

**Table 4:** The mean difference of TF/IDF of PT perspective between high (1,2) and low (4,5) quintile.

Variable	High			Low			Difference	*t*-Value	Prob-t	Significance
	N	Mean	Std	N	Mean	Std				
SIZE	27	25.387	1.881	26	25.119	1.682	0.268	0.55	0.587	.
ROA	27	0.044	0.140	26	−0.013	0.120	0.057	1.60	0.115	.
FTOV	27	3.949	4.546	26	6.526	10.747	−2.577	−1.13	0.267	.
LTLEV	27	0.476	0.668	26	0.352	0.436	0.124	0.80	0.427	.
CUR	26	1.260	1.022	26	1.314	0.868	−0.054	−0.21	0.838	.
ACCRV	26	0.738	0.977	26	0.286	0.822	0.452	1.82	0.075	*

Note: * denotes 0.10 significance level.

**Table 5:** The mean difference of TF/IDF of IP perspective between high (1,2) and low (4,5) quintile.

Variable	High			Low			Difference	*t*-Value	Prob-t	Significance
	N	Mean	Std	N	Mean	Std				
SIZE	26	24.354	1.788	26	25.687	1.322	−1.332	−3.06	0.004	***
ROA	26	0.039	0.170	26	−0.014	0.072	0.052	1.45	0.156	.
FTOV	26	7.134	9.282	26	4.801	8.277	2.333	0.96	0.343	.
LTLEV	26	0.542	0.786	26	0.396	0.430	0.146	0.83	0.411	.
CUR	26	1.567	1.471	26	1.298	1.053	0.269	0.75	0.455	.
ACCRV	26	0.405	0.711	26	0.600	0.751	−0.195	−0.97	0.336	.

Note: *** denotes 0.01 significance level.

**Table 6:** The mean difference of TF/IDF of LG between high (1,2) and low (4,5) quintile.

Variable	High			Low			Difference	*t*-Value	Prob-t	Significance
	N	Mean	Std	N	Mean	Std				
SIZE	26	25.569	1.725	26	24.945	1.879	0.623	1.25	0.219	.
ROA	26	−0.019	0.061	26	0.028	0.173	−0.047	−1.31	0.200	.
FTOV	26	3.179	3.180	26	8.847	11.537	−5.668	−2.42	0.022	**
LTLEV	26	0.341	0.329	26	0.642	0.807	−0.301	−1.76	0.087	*
CUR	26	1.560	1.405	26	1.365	1.159	0.195	0.55	0.588	.
ACCRV	26	0.385	0.598	26	0.814	0.982	−0.430	−1.92	0.063	*

Note: ** and * denote significance at 0.05 and 0.10 levels, respectively.

## Data Availability

Data could be obtained from KIS-Line and KIS-Value in NICE Information database, which is available at https://www.kisvalue.com/web/index.jsp (accessed on 1 April 2021).

## Appendix A

**Table A1:** The words categorized to balance scorecard.

Category Name	N of Words	Details
Public (PB)	16	Domestic, nation, contribution, Republic of Korea, resident, welfare, service, society, life, initiative, citizen, action, inhabitant, the first, vulnerability, happiness
Patient (PT)	15	home, family, appreciation, client, interest, origin, mind, visit, love, trust, appointment, use, information, support, sincerity
Internal Process (IP)	18	open, base, faculty, establishment, international, introduction, business, foundation, world, facilities, system, safety, high quality, role, management, equipment, cutting edge, securement
Learning and Growth (LG)	18	care, education, change, ward, sickbed, sanitation, study, medical personnel, medicine, specialty, spirit, enhancement, continuation, disease, illness, high-tech, treatment, environment
Neutral (NEU)	13	class, national, university hospital, basis, level, performance, beginning, history, we, best, the best, the latest, the present
